# US in the assessment of acute scrotum

**DOI:** 10.1186/2036-7902-5-S1-S8

**Published:** 2013-07-15

**Authors:** Alfredo D’Andrea, Francesco Coppolino, Elviro Cesarano, Anna Russo, Salvatore Cappabianca, Eugenio Annibale Genovese, Paolo Fonio, Luca Macarini

**Affiliations:** 1 S G. Moscati Hospital, Department of Radiology, Aversa, Italy; 2 University of Palermo, Department of Radiology, Palermo, Italy; 3 Radiology Section. Health service. Navy Command of Brindisi, Brindisi, Italy; 4 Second University of Naples, Department of Clinical and Experimental Internistic F. Magrassi, Naples, Italy; 5 University of Cagliari, Department of Radiology, Cagliari, Italy; 6 Institute of Diagnostic and Interventional Radiology, University of Turin , Turin Italy; 7 Department of Radiology, University of Foggia, Foggia, Italy

**Keywords:** scrotal trauma, scrotal disease, ultrasound of scrotum

## Abstract

**Background:**

The acute scrotum is a medical emergency . The acute scrotum is defined as scrotal pain, swelling, and redness of acute onset. Scrotal abnormalities can be divided into three groups , which are extra-testicular lesion, intra-testicular lesion and trauma. This is a retrospective analysis of 164 ultrasound examination performed in patient arriving in the emergency room for scrotal pain.

The objective of this article is to familiarize the reader with the US features of the most common and some of the least common scrotal lesions.

**Methods:**

Between January 2008 and January 2010, 164 patients aged few month and older with scrotal symptoms, who underwent scrotal ultrasonography (US), were retrospectively reviewed. The clinical presentation, outcome, and US results were analyzed. The presentation symptoms including scrotal pain, painless scrotal mass or swelling, and trauma.

**Results:**

Of 164 patients, 125 (76%) presented with scrotal pain, 31 (19%) had painless scrotal mass or swelling and 8 (5%) had trauma. Of the 125 patients with scrotal pain, 72 had infection,10 had testicular torsion, 8 had testicular trauma, 18 had varicocele, 20 had hydrocele, 5 had cryptorchidism, 5 had scrotal sac and groin metastases, and 2 had unremarkable results. In the 8 patients who had history of scrotal trauma, US detected testicular rupture in 1 patients, scrotal haematomas in 2 patients .

Of the 19 patients who presented with painless scrotal mass or swelling, 1 6 had extra-testicular lesions and 3 had intra-testicular lesions. All the extra-testicular lesions were benign. Of the 3 intra-testicular lesions, one was due to tuberculosis epididymo-orchitis, one was non-Hodgkin’s lymphoma, and one was metastasis from liposarcoma

**Conclusions:**

US provides excellent anatomic detail; when color Doppler and Power Doppler imaging are added, testicular perfusion can be assessed

## Background

The acute scrotum is a medical emergency defined as scrotal pain, swelling, and redness of acute onset [1-3]. The differential diagnosis includes torsion, infection, trauma, tumor, and other rarer causes. The diagnostic evaluation begins with history-taking. Scrotal abnormalities can be divided into three groups , which are extra-testicular lesion, intra-testicular lesion and trauma. Causes of scrotal pain include inflammation (epididymitis, epididymo-orchitis, abscess), testicular torsion, testicular trauma, and testicular cancer [4-12].

Prompt diagnosis is required to differentiate surgically correctable lesions from abnormalities that can be adequately treated by medical therapy alone. Clinical symptoms and physical examination are often not enough for definite diagnosis due to pain and swelling that limit an accurate palpation of the scrotal contents.

For patients presenting with a scrotal mass, it is critical to determine whether the mass is intra- or extra-testicular. This is important because the majority of intra-testicular lesions are malignant, while extra-testicular lesions are usually benign.

US provides excellent anatomic detail; when color Doppler and power Doppler imaging are added, testicular perfusion can be assessed.

The objective of this article is to familiarize the reader with the US features of the most common and some of the least common scrotal lesions.

This is a retrospective analysis of 164 ultrasound examination performed in patient arriving in the emergency room for scrotal pain. Between January 2008 and January 2010, 164 patients aged few month to 80 years with scrotal symptoms, who underwent scrotal ultrasonography (US), were retrospectively reviewed.

## Methods

Between January 2008 and January 2010, 164 patients aged few month and older with scrotal symptoms, who underwent scrotal ultrasonography (US), were retrospectively reviewed. The clinical presentation, outcome, and US results were analyzed. The presentation symptoms including scrotal pain, painless scrotal mass or swelling, and trauma.

The patients have performed an ultrasound examination positioned supine, and a rolled towel or sheet is placed between the legs to support the scrotum. The penis is displaced superiorly or super-laterally with a towel draped over it. Scanning is performed with a high-frequency (8–15-MHz) transducer in sequential sagittal and transverse planes.

In cases of marked scrotal enlargement, we used a lower frequency transducer. Scanning of both testes is performed in sagittal and transverse planes with size measurements. Transverse side-by-side images of both testes should be obtained for comparison of echo texture, skin thickness, and color Doppler flow pattern. The epididymis should be imaged on the long and short axes. Color and power Doppler imaging are used to detect flow within the scrotal structures and to confirm symmetric or abnormal flow patterns.

## Results and discussion

Of 164 patients, 125 (76%) presented with scrotal pain, 31 (19%) had painless scrotal mass or swelling and 8 (5%) had trauma. Of the 125 patients with scrotal pain, 72 had infection, 10 had testicular torsion, 8 had testicular trauma, 18 had varicocele, 20 had hydrocele, 5 had cryptorchidism, 5 had scrotal sac and groin metastases, and 2 had unremarkable results. In the 8 patients who had history of scrotal trauma, US detected testicular rupture in 1 patients, scrotal haematomas in 2 patients .

The patients with ruptured testes underwent operation of partial orchiectomy .

Of the 19 patients who presented with painless scrotal mass or swelling, 16had extra-testicular lesions and 3 had intra-testicular lesions. All the extra-testicular lesions were benign. Of the 3 intra-testicular lesions, one was due to tuberculosis epididymo-orchitis, one was non-Hodgkin’s lymphoma, and one was metastasis from liposarcoma.

The acute scrotum is a medical emergency [1,2]. The acute scrotum is defined as scrotal pain, swelling, and redness of acute onset. The differential diagnosis includes torsion, infection, trauma, tumor, and rarer causes. The diagnostic evaluation begins with history-taking. The patient should be asked about the exact temporal course of events, the intensity of the pain, and, in particular, when the pain began and in the trauma what is the traumatic mechanism (blunt, penetrating, degloving, and electrical burn injuries to scrotal contents). Trauma often may result in hematoma, hydrocele, hematocele, testicular fracture, or testicular rupture.

If the patient is a very small child, this information can only be obtained from a parent. The physician must also ask any new systemic symptoms or diseases already known to be present [1,2,13], particularly local problems, such as an inguinal hernia. symptoms of hematologic disease and any newly arisen hematoma or petechiae should be asked about specifically. On physical examination, the scrotum should be inspected, and a brief general physical examination should be performed [13]. The involved testis should be palpated, and its position, size, and tenderness (if any) should be noted in comparison to the other side. The testis and epididymis should be evaluated separately, if possible. Next, the inguinal canal and the abdomen are palpated, and the cremasteric reflex is tested. When a patient presents with scrotal trauma, a clinical assessment is made for acute scrotal pain, swelling, and bruising. The overlying skin is examined to determine the extent of its integrity and for sites of any entry and exit wounds. The testis and epididymis also are palpated, and a penile examination is performed by the clinician. After any necessary wound cultures, urinalysis, and urine cultures are performed, the patient undergoes imaging with US unless there is scrotal avulsion [14,15].

Scrotal abnormalities can be divided into three groups, which are extra-testicular lesion, intra-testicular lesion and trauma. Causes of scrotal pain include inflammation (epididymitis, epididymo-orchitis, abscess), testicular torsion, testicular trauma, and testicular cancer [5-16].

Prompt diagnosis is required to differentiate surgically correctable lesions from abnormalities that can be adequately treated by medical therapy alone[17-19]. Clinical symptoms and physical examination are often not enough for definite diagnosis due to pain and swelling that limit an accurate palpation of the scrotal contents [9,10]. For patients presenting with a scrotal mass, it is critical to determine whether the mass is intra- or extra-testicular. This is important because the majority of intra-testicular lesions are malignant, while extra-testicular lesions are usually benign.

High resolution ultrasonography (US) combined with Colour Doppler ultrasonography (CDUS) has become the imaging modality of choice for evaluating scrotal diseases [1]. US is helpful in differentiating extra- from intra-testicular lesions [9,10]. Ultrasonography (US) is commonly performed for the assessment of scrotal abnormalities. It is ideal for the assessment of scrotal trauma, as it can be used for noninvasive evaluation of the scrotal contents, testicular integrity, and blood flow, as well as to visualize hematomas, other fluid collections, and foreign bodies.

**Figure 1:**
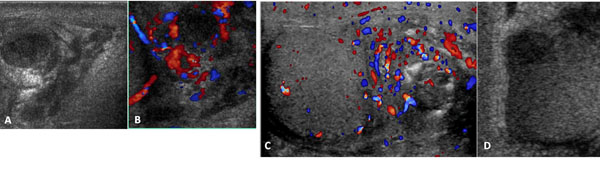
(a,b,c,d) Gray-scale and Colour Doppler of epididymis US findings of these lesions, including enlarged epididymis and/or testis with heterogeneous echogenicity, are overlapping but CDUS findings are different (a). The inflamed epididymis and testis have increased blood flow whereas testicular torsion has decreased blood flow(b). The epididymal head is the most affected region, and reactive hydrocele and wall thickening are frequently present(c). Increased size and, depending on the time of evolution, decreased, increased, or heterogeneous echogenicity of the affected organ are usually observed (d).

We found 72 cases of epididymo-orchitis mean age 50.9 . The most common cause of scrotal pain is infection , which was mostly found in middle-aged men. Epididymitis and orchitis are either viral or bacterial infections of the epididymis and testis. Bacterial infections are very rare in children, unlike in adults. The infection usually originates in the bladder or prostate gland, spreads through the vas deferens and the lymphatics of the spermatic cord to the epididymis, and finally reaches the testis, causing epididymo-orchitis. Isolated orchitis is very rare. The clinical spectrum ranges from mild tenderness to a severe febrile process. Bacterial epididymitis or epididymo-orchitis are the most common causes of scrotal pain in adults while torsion is more common in a younger age group [9-11,16]. Gray-scale US findings of these lesions, including enlarged epididymis and/or testis with heterogeneous echogenicity, are overlapping but CDUS findings are different. The inflamed epididymis and testis have increased blood flow whereas testicular torsion has decreased blood flow. The epididymal head is the most affected region, and reactive hydrocele and wall thickening are frequently present. Increased size and, depending on the time of evolution, decreased, increased, or heterogeneous echogenicity of the affected organ are usually observed (Figure 1). The inflammation produces increased blood flow within the epididymis, testis, or both.

We found 10 patient with testicular torsion with mean age of 26 years. Six of these patients presented early, between 1 and 4 hours after the onset of pain. US gave correct diagnosis leading to prompt surgical correction and the testes were salvaged.

**Figure 2:**
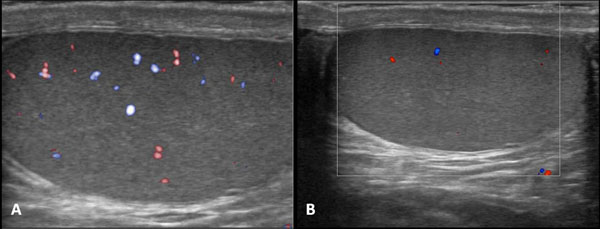
(a,b) Gray-scale and Colour Doppler of testicular torsion. In the early phases of torsion (1–3 hours), testicular echogenicity appears normal. With progression, enlargement of the affected testis and increased or heterogeneous echogenicity are common findings. A definitive diagnosis of complete testicular torsion is made when blood flow is visualized on the normal side but is absent on the affected side .Incomplete torsion refers to cord twisting of less than 360°, in which some arterial flow persists in the affected testis .

The other 4 patients came late, between 3 and 6 days after their symptoms appeared. Orchiectomy was performed after diagnosis of missed torsion. Testicular torsion is a suddenly occurring rotation of a testis about its axis, resulting in twisting of the spermatic cord. The pain is acute and severe and may be accompanied by vomiting; shock-like symptoms are rare. Because the testicular parenchyma cannot tolerate ischemia for more than a short time, testicular torsion must be ruled out rapidly as the cause. Testicular torsion accounts for about 25% cases of acute scrotum, with an incidence of roughly 1 per 4000 young males per year. Testicular torsion, or twisting of the spermatic cord, implies first venous and later arterial flow obstruction. The extent of testicular ischemia will depend on the degree of twisting (180°–720°) and the duration of the torsion. Testicular salvage is more likely in patients treated within 4–6 hours after the onset of torsion. On the basis of the surgical findings, two types of testicular torsion are recognized: extra-vaginal and intra-vaginal. Extra-vaginal torsion is seen mainly in newborns and occurs prenatally in most cases [17,18]. The testis is usually necrotic at birth and the hemiscrotum is swollen and discolored. US findings vary, but complex hydrocele and calcification of the tunica albuginea are common. Intra-vaginal torsion can occur at any age but is more common in adolescents. A predisposing factor is the “bell clapper” deformity, in which the tunica vaginalis joins high on the spermatic cord, leaving the testis free to rotate. Differentiation between testicular torsion and epididymo-orchitis is a clinical challenge, since scrotal pain, swelling, and redness or tenderness are clinical symptoms common to these two entities. The usual teaching is that pain in testicular torsion has a sudden onset, whereas in orchitis it is more gradual. In the early phases of torsion (1–3 hours), testicular echogenicity appears normal. With progression, enlargement of the affected testis and increased or heterogeneous echogenicity are common findings. Sonographic evaluation of the spermatic cord is an essential part of the examination. The point of cord twisting can be identified at the external inguinal orifice . The intra-scrotal portion of the edematous cord appears as a round, ovoid, or curled echogenic extra-testicular mass, with the epididymal head wrapped around it . The orientation of the testis, epididymis, and cord may be inverted [19-22]. A definitive diagnosis of complete testicular torsion is made when blood flow is visualized on the normal side but is absent on the affected side .Incomplete torsion refers to cord twisting of less than 360°, in which some arterial flow persists in the affected testis (Figure 2). Meticulous comparison of the two testes by using transverse views is mandatory in these cases.

We found five patient with undescended testes (mean age 4,5). Failure of the intra-abdominal testes to descend into the scrotal sac is known as cryptorchidism. The cryptorchid testis may be located at any point along the descent route. The prevalence of this condition is 9.2%–30% in premature infants and 3.4%–5.8% in full-term infants [23]. The testes originate within the retro-peritoneum and migrate downward through the internal inguinal ring, inguinal canal, and external inguinal ring to the scrotum. Sonography is useful only for identifying testes in the inguinal canal (the most frequent location ) or the pre-scrotal region just beyond the external inguinal ring and should be the initial imaging procedure. The cryptorchid testis is usually smaller and isoechoic or hypoechoic relative to the normally located testis.

We found in our analysis 18 patient with varicocele (mean age 30.6).and 20 with hydrocele (mean age 40); 6 hydroceles in our study are associated with inflammatory process, 6 with testicular torsion, 5 with trauma, and 3 with tumor.

Varicocele involves abnormal dilatation of veins in the pampiniform plexus of the spermatic cord and is relatively common.

Most cases are idiopathic; varicoceles are found mainly in adolescents and young adults and are more frequent on the left side [24].At sonography, the dilated veins appear as tortuous, anechoic, tubular structures along the spermatic cord. On color and pulsed-wave Doppler images, venous flow is better demonstrated during the Valsalva maneuver. Varicocele may affect testicular growth; hence, testicular volumes should be systematically measured and asymmetries assessed with US.

Hydrocele, an abnormal collection of fluid between the visceral and parietal layers of the tunica vaginalis and/or along the spermatic cord, is the most common cause of painless scrotal swelling in children. In the normal scrotum, 1–2 mL of serous fluid may be observed in the potential tunica vaginalis cavity and should not be mistaken for hydrocele. Virtually all hydroceles are congenital in neonates and infants and associated with a patent processus vaginalis, which allows peritoneal fluid to enter the scrotal sac [25]. Up to 50% of acquired hydroceles are due to trauma [26], and hydroceles may occur in up to 25% of patients with major trauma [27].

We found 5 inguinal hernia (middle age 26.7). Inguinal-scrotal hernia is defined as the passage of intestinal loops and/or omentum into the scrotal cavity. The prevalence of inguinal hernia is higher in preterm neonates, especially at 32 weeks gestation ; in our experience two patient are three month [28]. The hernia is more frequently located on the right side, since the right processus vaginalis closes later than the left [29]. Physical examination is sufficient to enable diagnosis in most cases. Nevertheless, US examination (which has replaced plain radiography) is indicated in patients with inconclusive physical findings, in patients with acute scrotum, and to investigate contralateral involvement in patients in whom only a unilateral hernia is clinically evident [30]. At gray-scale US, the scrotum is partially occupied by one or more round structures containing air bubbles or fluid . The diagnosis of hernia is achieved by visualization of air bubble movement and/or intestinal peristalsis during the real-time examination. The herniated omentum is seen as a highly echogenic structure. Inguinal rings larger than 4 mm are an indication for prophylactic herniorrhaphy [31-34]).We routinely use color or power Doppler imaging in inguinal-scrotal hernia to investigate intestinal and testicular perfusion. We found four patients with an akinetic dilated bowel loop (a sign of strangulation) and impaired testicular perfusion.

In the our study we analyzed 10 patient with trauma; 8 patients with blunt injuries (sporting activities and motocyclistic trauma) 5 associated with hematocele and 3 associated with hematoma; 2 patients with penetrating injuries (one of knives lesion, and one of animal bites). Blunt injuries are noninvasive injuries from high energy transferred during contact with a solid object (eg, from a kick to the groin). The main mechanism of injury in blunt trauma is crushing of the testis against the symphysis pubis or between the thighs. Penetrating injuries include wounds from sharp objects and missiles (eg, knives, bullets) as well as animal bites and injuries from self-mutilation.

Hematoceles is similar to hydroceles, hematoceles are complex collections that separate the visceral and parietal layers of the tunica vaginalis. Like hematomas, they are acutely echogenic and become more complex and more hypoechoic with age. Subacute and chronic hematoceles may contain fluid-fluid levels or low-level internal echoes. Hematomas may occur in intra-testicular locations or in extra-testicular soft tissues such as the scrotal wall or epididymis. The lesions are usually focal, may be multiple, and may be hyperechoic (in acute bleeding) or hypoechoic (as the hemorrhage ages) and lack vascularity . The fluid in a complex hematoma may be heterogeneous. Hematomas of the scrotal wall may appear as focal thickening of the wall or as fluid collections within the wall.

A testicular fracture appears as a linear hypoechoic band that extends across the testicular parenchyma and represents a break in the normal testicular architecture . The overall contour remains smooth, as the testicular shape and the tunica albuginea are maintained. Doppler imaging is used to determine vascular integrity.Testicular fractures are treated conservatively if normal flow is identified. If flow is absent, emergent surgery is indicated, as this finding represents ischemia [35,36].

**Figure 3:**
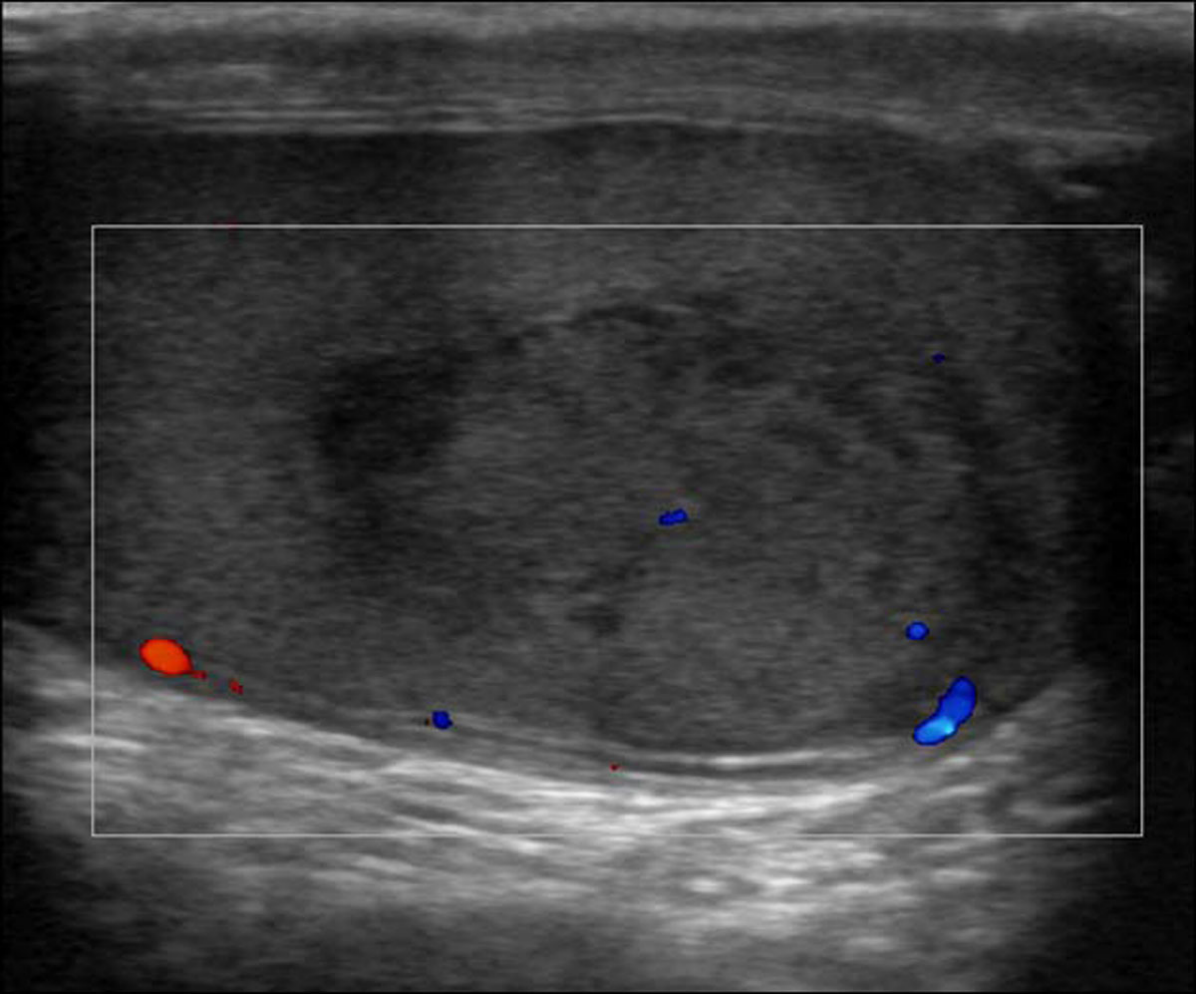
Gray scale and Colour Doppler of testicular trauma. In this type of injury, US images also demonstrate poorly defined testicular margins and heterogeneous echotexture, with focal hyperechoic or hypoechoic areas in the testicular parenchyma corresponding to areas of hemorrhage or infarction . Color and duplex Doppler images may show decreased flow or no flow.

In testicular rupture, there is hemorrhage and extrusion of testicular contents into the scrotal sac [37]. Discontinuity of the echogenic tunica albuginea is indicative of testicular rupture and necessitates emergent surgery. In this type of injury, US images also demonstrate poorly defined testicular margins and heterogeneous echotexture, with focal hyperechoic or hypoechoic areas in the testicular parenchyma corresponding to areas of hemorrhage or infarction [38,39]. Associated findings may include scrotal wall thickening and hematocele. Color and duplex Doppler images may show decreased flow or no flow (Figure 3).

## Conclusions

Ultrasonography b-mode and colour Doppler ultrasonography has become the imaging modality of choice for evaluating acute scrotal diseases in emergency room. Ultrasound, is a valid method for the study and for the immediate diagnosis in the emergency room of these pathologies.

## Competing interests

The authors declare that they have no competing interests.

## Declarations

This article has been published as part of *Critical Ultrasound Journal* Volume 5 Supplement 1, 2013: Topics in emergency abdominal ultrasonography. The full contents of the supplement are available online at http://www.criticalultrasoundjournal.com/supplements/5/S1. Publication of this supplement has been funded by the University of Molise, University of Siena, University of Cagliari, University of Ferrara and University of Turin.
